# Flexible, photoluminescent 0D Cs_4_PbX_6_ (X = Br, Br/I)–PMMA composite films for white LED via water-induced recrystallization

**DOI:** 10.1557/s43578-024-01326-4

**Published:** 2024-05-16

**Authors:** Yuang Ji, Shihai Wang, Haohan Yang, Donghai Lin, Wan Y. Shih, Wei-Heng Shih

**Affiliations:** 1 Shanghai Key Laboratory of Engineering Materials Application and Evaluation, Shanghai Thermophysical Properties Big Data Professional Technical Service Platform, Shanghai Engineering Research Center of Advanced Thermal Functional Materials, School of Energy and Materials, Shanghai Polytechnic University, Shanghai, 201209 China; 2 School of Biomedical Engineering, Science, and Health Systems, Drexel University, Philadelphia, USA; 3 Department of Materials Science and Engineering, Drexel University, Philadelphia, USA

**Keywords:** Inorganic halide perovskite, Cs_4_PbX_6_, Photoluminescence, PMMA, Stability, LED

## Abstract

3D CsPbX_3_ inorganic perovskite materials have attracted much attention in optoelectronic devices because of their strong absorbance, high photoluminescent quantum yield, tunable band gap, and narrow emission bandwidth. However, their practical usefulness is limited due to their poor stability in ambient conditions. Here, we created photoluminescent 0D Cs_4_PbX_6_ (X = Br, Br/I) suspensions in toluene by adding a small amount of water. The photoluminescent 0D Cs_4_PbX_6_ perovskite was mixed with polymethylmethacrylate (PMMA) forming 0D Cs_4_PbX_6_/PMMA composite films with higher PL, stability, transparency, and transmittance than that of the 3D CsPbX_3_/PMMA composite films prepared separately. Moreover, the PL intensity maintains 90% of the initial value after 30 days in water, showing excellent water stability. The flexible white-light LED device prepared by the composite films illustrated good luminescence performance with color rendering index 74.77, chromaticity coordinates (0.32, 0.33), and color temperature 6997 K.

**Graphical abstract:**

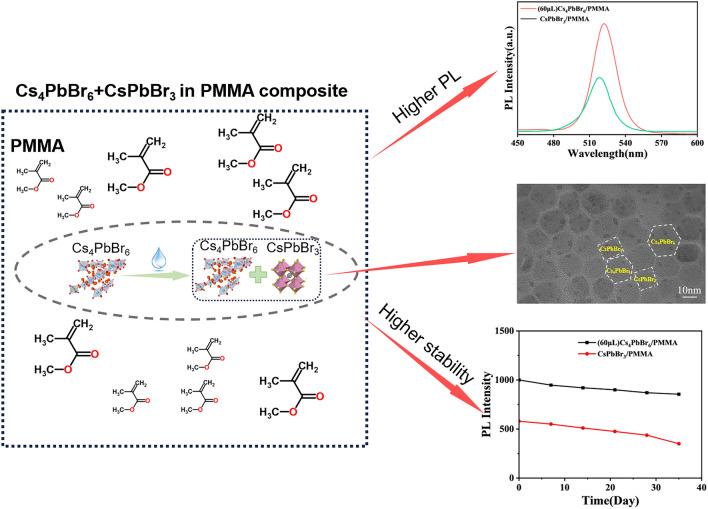

## Introduction

In recent years, inorganic halide perovskites have shown great potential as a new type of optoelectronic material in solar cells [1], photocatalysis [2], and optoelectronic devices [3–7] due to their excellent optical properties and low cost. Although its high color purity, high carrier mobility, and long carrier diffusion length [8–10] are very attractive, its crystal structure is easily degraded from cubic to the orthorhombic structure at room temperature [11], and the lack of stability in water [12], oxygen [13], and elevated temperature [14] hinder its practical usefulness. Researchers tried to explore other structures of halide perovskites with better properties. Halide perovskites have a structure characterized by how the (PbX_6_)^4−^ octahedra are shared. When all corners of (PbX_6_)^4−^ octahedra are shared forming a 3D network of octahedra, namely 3D CsPbX_3_, the splitting of the bonding and antibonding states results in much smaller band gaps with photoluminescence in the visible light range. Meanwhile, the individual (PbX_6_)^4−^ without sharing corners has a structure called 0D Cs_4_PbX_6_ and maintains a large band gap. Saidaminov et al. synthesized 0D Cs_4_PbBr_6_ powders with bright green luminescence by rapid precipitation from CsBr/PbBr_2_ (1:1 molar ratio) in dimethyl sulfoxide (DMSO) solution by adding antisolvent (dichloromethane), followed by washing with DMSO [15]. Their unique broadband emission and large Stokes shift make them good candidates for white LED devices. However, Akkerman et al. [16] made 0D Cs_4_PbBr_6_ by injecting 150 °C Cs oleate precursor into a PbX_2_ solution in octadecene containing oleic acid and oleylamine. They found the 0D Cs_4_PbBr_6_ synthesized by the hot injection method was not photoluminescent with a large band gap. They argued that the photoluminescent Cs_4_PbBr_6_ reported in the literature was due to a small amount of CsPbBr_3_ embedded in the 0D Cs_4_PbBr_6_. On the other hand, Yang et al. [17] and Wang et al. [18] argued that the presence of intrinsic defect bromine vacancies and the introduction of hydroxyl groups may lead to a narrowing of the band gap and the formation of defect energy levels, resulting in PL emission. Therefore, water plays an important role in the photoluminescence of 0D Cs_4_PbBr_6_ and it is necessary to investigate the effect of water. In fact, it has been shown that 0D Cs_4_PbBr_6_ could become photoluminescent by adding a small amount of water [18, 19].

Although there have been some studies on 0D Cs_4_PbBr_6_ [20–22], there are few studies on 0D Cs_4_PbI_6_ which has broadband emission [23, 24]. Even though stability of 0D Cs_4_PbX_6_ is higher than that of 3D CsPbX_3_ [25], there is still need to protect 0D Cs_4_PbX_6_ to improve its stability for practical applications. Pinchetti et al. [26] reported that Mn-doped CsPb_x_Mn_1−x_I_3_ films could improve the stability by maintaining a black cubic α-CsPbI_3_ phase for one month, while undoped CsPbI_3_ thin film turned into the yellow δ-CsPbI_3_ phase within 5 days. Chen et al. [27] introduced SEBS elastomers to synthesize highly flexible and stable CsPbBr_3_/SEBS composite films which exhibited excellent water resistance, maintaining 94% of their initial strength after more than 110 days in water. Li et al. used PH-TMOS to create silanol groups on Cs_4_PbBr_6_ followed by hydrolysis to prepare highly luminescent CsPbBr_3_@SiO_2_ capsules. After 90 days in air, bright green emission was still observed under UV light [28]. Kar et al. [29] synthesized Zn-doped CsPbBr_3_ at room temperature using a modified ligand-assisted reprecipitation method. The obtained nanocrystals have 88% PLQY as well as optimized water stability by a factor of 4. The strategies to enhance the stability of 3D structures are also applicable to 0D structures. One example is polymer wrapping, which protects the perovskite nanocrystals by isolating the CsPbX_3_ nanocrystals from water, light, and oxygen. Another example is to grow CsPbBr_3_ in situ in metal–organic frameworks ZIF-8 and ZIF-67 and obtain the composite samples that remain stable in water for 10 days [30]. Due to the higher resistance to the environment, the polymer cladding material can effectively passivate the surface defects and modulate the optoelectronic properties of nanocrystals, while also significantly improving their stability in water and under oxygenated conditions. The outer layer protection can also greatly reduce the hydrolysis and agglomeration of the nanocrystals [31].

Water is damaging to halide perovskites normally. However, Zhang et al. [32] observed that a small amount of water added to toluene solvent in their LARP process [33] could help make CsPbBr_3_ in toluene brighter with an altered morphology. The idea was that a small amount of water dissolves CsPbBr_3_ nanocrystals and recrystallizes on top of the preexisting nanocrystals, making particles larger and changing their morphology. In this paper, we used polymethyl methacrylate (PMMA) as a matrix to protect the 0D Cs_4_PbBr_6_ by adding PMMA to the Cs_4_PbBr_6_ toluene suspensions with a small amount of water forming photoluminescent composite films. It was found that using a small amount of water can recrystallize photoluminescent CsPbBr_3_ embedded in PMMA making the Cs_4_PbBr_6_/PMMA composites photoluminescent.

In addition, we introduced smaller Br ions to partially replace I ions to synthesize 0D Cs_4_PbBr_2.4_I_3.6_ in toluene followed by water addition to create photoluminescence with bright red light. The obtained 0D Cs_4_PbBr_2.4_I_3.6_ in toluene suspensions possess higher PL intensity than 3D CsPbBr_3_ or CsPbBr_1.2_I_1.8_ suspensions. The 0D Cs_4_PbBr_2.4_I_3.6_ toluene suspension was also made into PMMA composites. The prepared composite films not only have high water stability but also have good transparency and refractive index. We also used composite films to prepare white-light LED which has excellent performance showing a good prospect for application.

## Experimental section

### Chemicals and materials

PbBr_2_ (99.9%), oleic acid (OA, 90.0%), oleamine (OAm, 90.0%), 1-octadecene (ODE, 90%), polymethylmethacrylate (PMMA), Cs_2_CO_3_(99.9%), and toluene (99.5%) were all purchased from Titan. All chemicals were used directly without further purification. The ultrapure water was used in all experimental procedures (18.2 MΩ cm).

### Preparation of Cs oleate

Typically, Cs_2_CO_3_ (0.407 g, 1.25 mmol), ODE (20 ml), and OA (1.2 ml) were mixed into a 50-ml three-neck flask and vacuum dried at 120 °C for 1 h. After the mixture was transferred to N_2_ and further heated to 150 °C, the temperature was maintained under stirring until all Cs_2_CO_3_ reacted with OA and the solution was clear. The prepared CsOA solution was stored at room temperature and pre-warmed to 100 °C before use.

### Preparation of 3D CsPbBr_3_ and CsPbI_3_ nanocrystals

PbBr_2_ (36.7 mg, 0.1 mmol), ODE (5 ml), OAm (0.5 ml), and OA (0.5 ml) were loaded into a 50-ml three-neck flask, vacuum heated to 120 °C for 1 h; the temperature was increased to 150 °C, and CsOA (0.3 ml) was quickly injected, and the solution was soaked in an ice water bath after waiting for 7 s and cooled to room temperature. The solution was centrifuged at 8000 rpm for 8 min, and the pellets were removed and dispersed in 5 ml toluene. For CsPbI_3_ nanocrystals, the amount of PbBr_2_ was replaced with PbI_2_ (46.1 mg, 0.1 mmol), and the reaction temperature was changed from 150 to 160 °C.

### Preparation of 0D Cs_4_PbBr_6_ and Cs_4_PbBr_2.4_I_3.6_ nanocrystals

PbBr_2_ (36.7 mg, 0.1 mmol), ODE (5 ml), OAm (0.5 ml), and OA (0.5 ml) were loaded into a 50-ml three-necked flask, vacuum heated to 120 °C for 1 h, the temperature was raised to 140 °C, and CsOA (1.1 ml) was quickly injected, and the solution was soaked in an ice water bath after waiting for 7 s and cooled to room temperature. The solution was centrifuged at 8000 rpm for 8 min, and the pellets were removed and dispersed in 5 ml toluene. For Cs_4_PbBr_2.4_I_3.6_ nanocrystals, the amount of PbBr_2_ was replaced with PbBr_2_ (14.68 mg, 0.04 mmol) and PbI_2_ (27.66 mg, 0.06 mmol), and the reaction temperature was changed from 140 to 150 °C.

### Preparation of CsPbBr_3_/PMMA and CsPbI_3_/PMMA composite films

Five ml of toluene was added to 2 g PMMA and stirred at 40 °C for 48 h until PMMA was completely dissolved. Five ml of previously prepared Cs_4_PbBr_6_ toluene solution was added, stirred, and mixed thoroughly to the PMMA solution for 2 h. One ml of the mixed solution was pipetted and filled into a quartz glass mold (3.8 cm × 1.2 cm × 0.1 cm). The excess solution outside of the mold was scraped and the film was dried at room temperature to obtain the CsPbBr_3_/PMMA composite films. For CsPbI_3_/PMMA composite films, the CsPbBr_3_ solution was replaced with the CsPbI_3_ solution.

### Preparation of Cs_4_PbBr_6_/PMMA and Cs_4_PbBr_2.4_I_3.6_/PMMA composite films

Five mL of toluene was added to 2 g PMMA and stirred at 40 °C for 48 h until PMMA was completely dissolved. Five ml of previously prepared Cs_4_PbBr_6_ toluene suspension was added with 60 μl of water. After ultrasonication of the Cs_4_PbBr_6_ suspension, the above PMMA toluene solution was added, followed by stirring for 2 h. One ml of the mixed solution was pipetted and filled into the quartz mold followed by drying at room temperature to form the composite films. For Cs_4_PbBr_2.4_I_3.6_/PMMA composite films, the Cs_4_PbBr_6_ solution was replaced with the Cs_4_PbBr_2.4_I_3.6_ solution.

### Preparation of WLED

A Cs_4_PbBr_6_/PMMA composite film and a Cs_4_PbBr_2.4_I_3.6_/PMMA composite film were stacked sequentially on a blue LED base with an operating current of 20 mA, sealed with black silicone and cured at room temperature for 30 min to form a white LED device.

### Characterization

The photoluminescence intensity and stability of the film were measured using a fluorescence spectrophotometer (Shimadzu RF-5301, Japan). UV absorbance was measured using a UV spectrophotometer (Shimadzu, Japan). Transmission electron microscopy (TEM) images were taken using a JEOL JEM-2100F 200 kV transmission electron microscope. The crystal structure of composite films was obtained from an X-ray diffractometer (XRD, D8 Advance, Bruker AG). The WLED was measured using EVERFINE's PCE-2000B single LED/mode photochromic electrical test system. The system consists of HASS-2000 high-precision fast spectral radiometer, LED300 test power supply, and integrating sphere for white LED testing.

## Results and discussion

**Figure 1:**
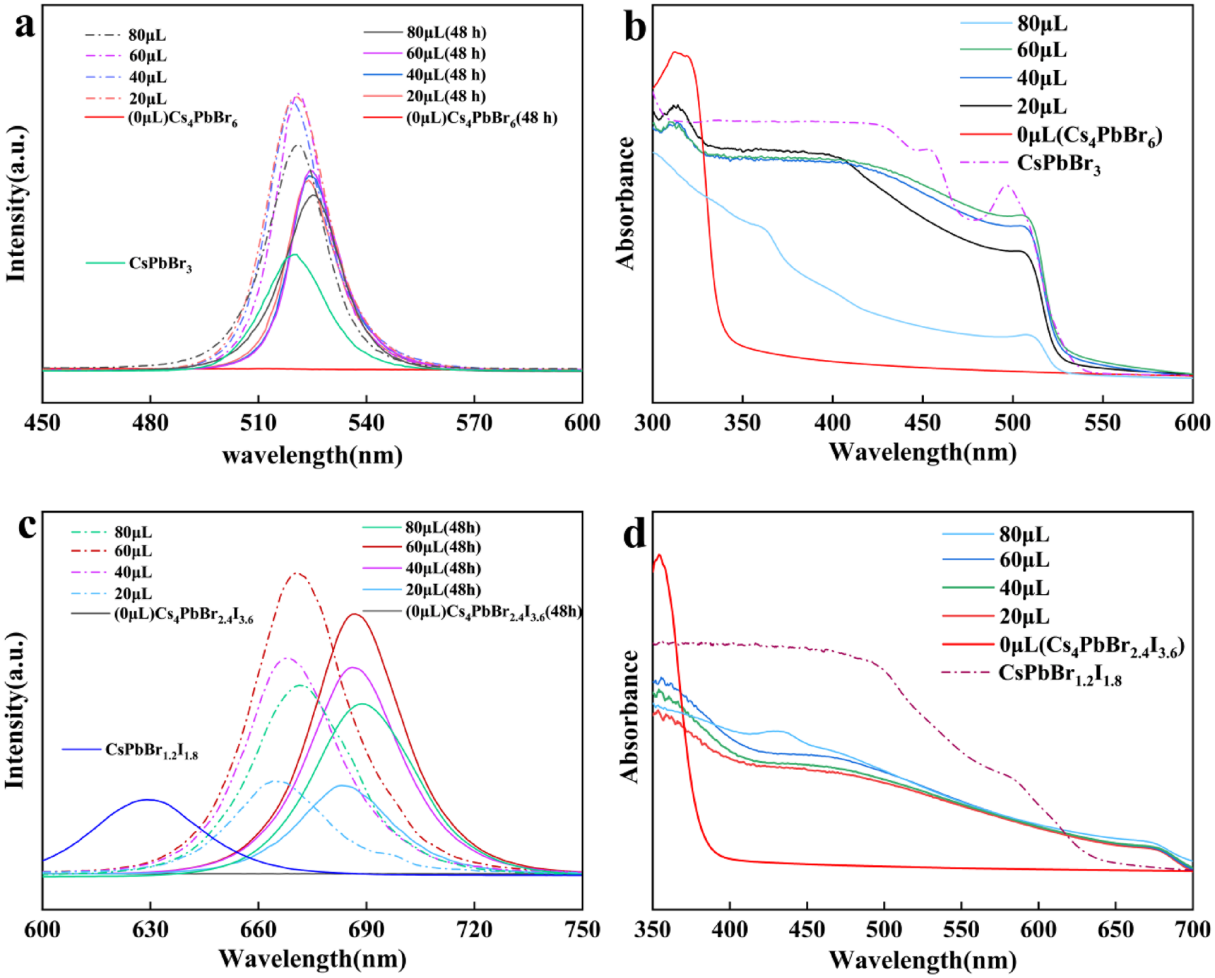
(a) PL spectra of Cs_4_PbBr_6_ NCs; (b) absorption spectra of Cs_4_PbBr_6_ NCs; (c) PL spectra of Cs_4_PbBr_2.4_I_3.6_ NCs; (d) absorption spectra of Cs_4_PbBr_2.4_I_3.6_ NCs, with different amounts of water.

To determine the specific effect of water on perovskite nanocrystals and select the best samples to prepare composite films, we added 20, 40, 60, and 80 μl of deionized water to 5 ml of 10 mM Cs_4_PbX_6_ toluene suspensions, respectively. The PL and absorption spectra were then measured. As shown in Fig. 1(a), in the Br system, 0D Cs_4_PbBr_6_ synthesized by the thermal injection method without the addition of water did not show PL emission under 350 nm UV excitation light. With the addition of water, part of the CsBr was dissolved and recrystallized to form 3D CsPbBr_3_ [19]. The photoluminescence peak was near 520 nm, similar to the peak position of CsPbBr_3_ synthesized by a separate thermal injection method. However, there was a small red shift of the PL peak of the emission peak position of the luminescent 0D Cs_4_PbBr_6_ compared to that of 3D CsPbBr_3_. Furthermore, the more water added, the larger the red shift of the PL peak was observed, consistent with the results of Zhang et al. [32] on the effect of water on CsPbBr_3_ in toluene. The PL intensity increased with the amount of water added and reached a peak at 60 μl water, and then decreased at 80 μl water, indicating the effect of water has exceeded a threshold which is similar to the results of Wang et al. [18] Interestingly, we observed that the red shift of PL peak increased with time, and the sample added with 80 μl water red shifted from 520 to 527 nm after 48 h. The red shift in PL wavelength is also observed as red shift in the absorption peak wavelength. As can be seen from Fig. 1(b), with the addition of water, the absorption peak representing Cs_4_PbBr_6_ at 314 nm began to decrease, and the absorption peak at 525 nm representing CsPbBr_3_ began to appear.

Figures 1(c) and d are the PL spectra and absorption spectra of the Br/I system after adding water. The absorption peak representing Cs_4_Pb_2.4_Br_3.6_ at 355 nm decreased, and at the same time a new absorption peak near 685 nm appeared. This indicates the completion of the recrystallization process of 3D CsPbBr_3−*x*_I_*x*_, and at the same time a photoluminescent peak appeared in the PL spectrum. The difference between the Br/I system and the Br system is that the PL peak of the recrystallized product of 0D Cs_4_PbBr_2.4_I_3.6_ is at 665 nm, which is different from the 630 nm of the 3D CsPbBr_1.2_I_1.8_ phase prepared by the hot injection method, while the PL peaks of the 0D Cs_4_PbBr_6_ and 3D CsPbBr_3_ are similar. The reason is that as water was added to 0D Cs_4_PbBr_2.4_I_3.6_, some CsBr and CsI were dissolved and recrystallized to form 3D CsPbBr_3−*x*_I_*x*_ with an x value different from 1.8 that corresponds to the original Br:I = 2:3 of 0D Cs_4_PbBr_2.4_I_3.6_. The difference in PL peak of 0D Cs_4_PbBr_2.4_I_3.6_ and 3D CsPbBr_1.2_I_1.8_ may be caused by the different degree of dissolution of CsBr and CsI resulting in different Br/I ratio during the recrystallization process. By comparing the PL peak of 665 nm of the recrystallized CsPbBr_3−*x*_I_*x*_, 630 nm of 3D CsPbBr_1.2_I_1.8_, and 690 nm of CsPbI_3_, [34] we concluded that *x* > 1.8 indicating the dissolution of CsI is more than CsBr. The red shift of PL due to the water addition in the Br/I system seems to be stronger than that in the Br system. For the Br system, the PL wavelength red shifted from 520 nm at the time of water addition to 527 nm after 48 h. In contrast, the PL peak red shifted from 665 nm at the time of 60 μl water addition to 687 nm after 48 h in the sample 0D Cs_4_PbBr_2.4_I_3.6_.

**Figure 2:**
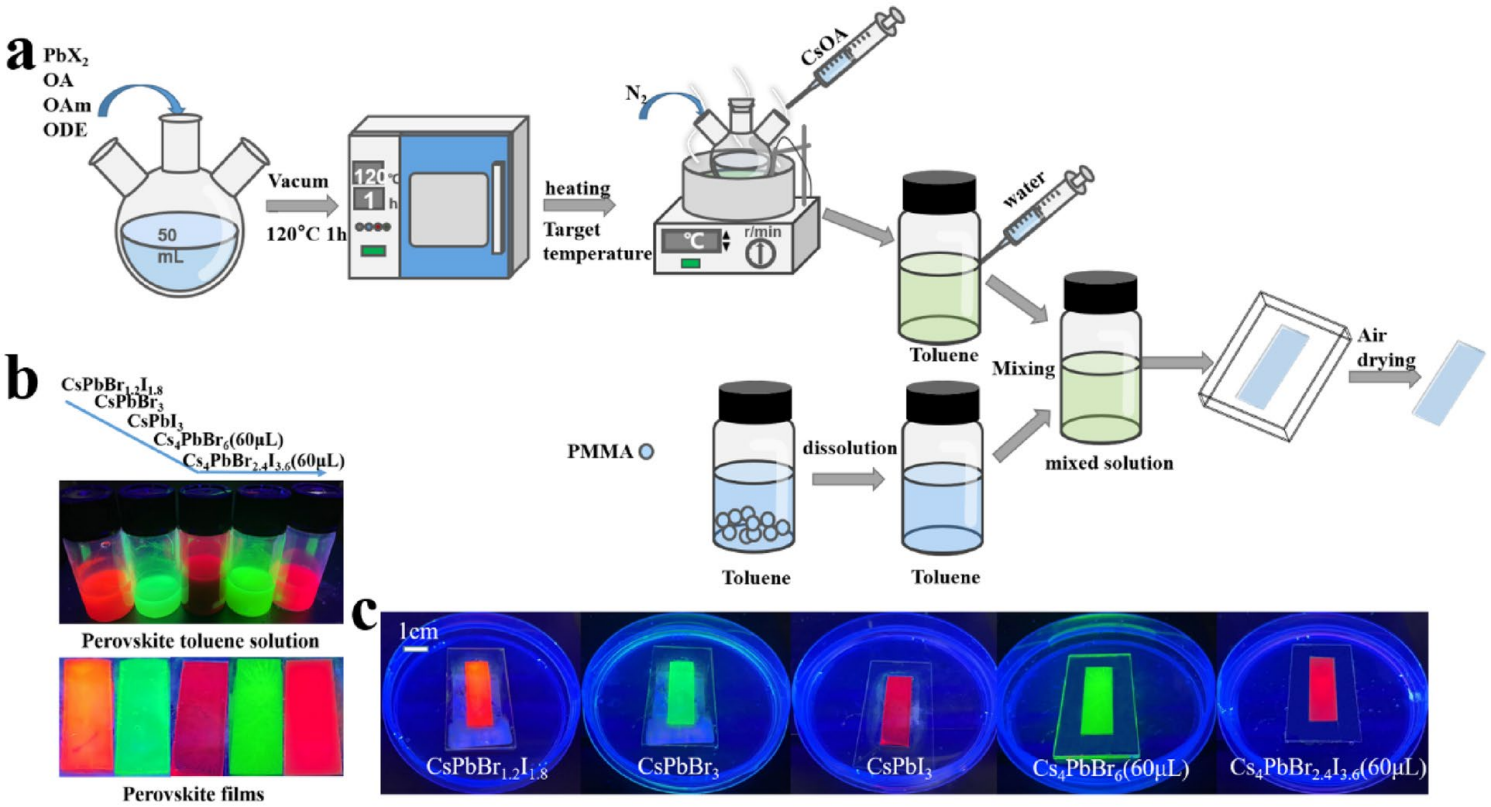
(a) Preparation process of composite film; (b) photographs of perovskite and PMMA complex toluene solution and perovskite–PMMA composite films under UV light; (c) photographs of perovskite–PMMA composite films in water under UV light.

The preparation procedure for the composite films is shown in Fig. 2(a) where we used the classical thermal injection method [13] for the synthesis of perovskites. After dissolving PbX_2_ in ODE at 140 °C, CsOA solution at 100 °C was injected, and 0D Cs_4_PbBr_6_ nanocrystals were precipitated through the process of nucleation and growth. The precipitates were collected and dispersed in toluene, followed by the addition of a small amount of water to the toluene solution. A separately prepared PMMA solution dissolved in toluene was mixed with the 0D Cs_4_PbBr_6_ toluene suspension and then filled into a mold of dimensions 3.8 cm long by 1.2 cm wide by 0.1 cm deep and dried in air to form a composite film. For the preparation of Br/I films, we prepared 0D Cs_4_PbBr_2.4_I_3.6_ with a PbBr_2_/PbI_2_ ratio of 2:3, and successfully obtained the red mixed halide films with high PL emission through the addition of water. The obtained composite films all have good luminescence properties. For comparison, we also prepared a 3D CsPbBr_3_ and CsPbBr_1.2_I_1.8_ perovskite composite films by the same method, respectively.

Figure 2(b) illustrates the physical images of the CsPbX_3_/PMMA and Cs_4_PbX_6_/PMMA composite films under 365 nm UV light, respectively. The perovskite nanocrystals were distributed uniformly giving out a uniform photoluminescence. In addition, Cs_4_PbBr_2.4_I_3.6_/PMMA composite film showed a brighter red to the naked eyes compared with that of the CsPbBr_1.2_I_1.8_/PMMA and CsPbI_3_/PMMA composite films. Figure 2(c) shows the physical pictures of the samples after 48 h in water. The stability of perovskite nanocrystals, which are extremely fragile in water and oxygen environment, has been greatly improved by the PMMA coating, indicating that the perovskite structure is protected. Furthermore, we found that the fluorescence brightness of the 3D CsPbBr_1.2_I_1.8_/PMMA composite films was reduced more than that of the 0D Cs_4_PbBr_2.4_I_3.6_/PMMA composite films.

**Figure 3:**
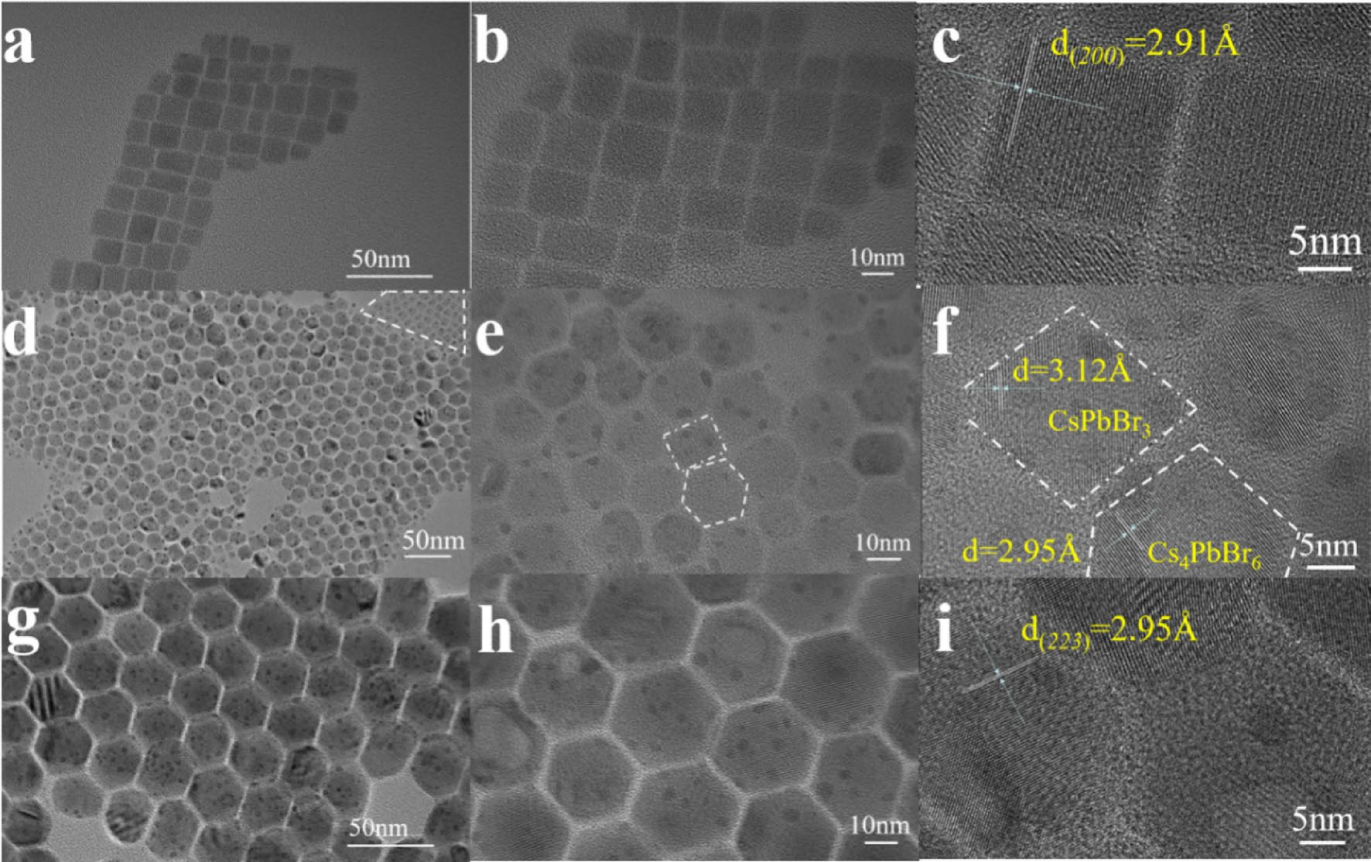
(a, d, g) The TEM, (b, e, h) the HR-TEM, and (c, f, and i) the magnified HR-TEM images of CsPbBr_3_, Cs_4_PbBr_6_ (with 60 μl water added), and Cs_4_PbBr_6_ NCs, respectively. The marked region in upper right corner of (d) indicates the aggregated square particles. The marked square and hexagon particles in (e) are analyzed in (f) for spacings of 3.12 Å and 2.95 Å, respectively.

The morphology and size distribution of perovskite nanocrystals were characterized and analyzed using transmission electron microscopy (TEM). 3D CsPbBr_3_ has cubic symmetry, and its crystal structure consists of angle-sharing PbBr_6_^4−^ octahedron. In Fig. 3(a, b), the CsPbBr_3_ images show a square shape. The nanoparticles are well dispersed, and the size range is 8–15 nm with an average size of 11 nm. The planar spacing in Fig. 3(c) was measured by Digital Micrograph to be 2.91 Å, which corresponds to the (200) crystal plane of the cubic phase CsPbBr_3_. In contrast, 0D Cs_4_PbBr_6_ consists of isolated [PbBr_6_]^4−^ octahedrons separated by Cs^+^ ions. Figure 3(g, h) shows the 0D Cs_4_PbBr_6_ NCs having a hexagon shape. The size range is 28–35 nm with an average size of 31 nm. The planar spacing was found to be 2.95 Å in Fig. 3(i) corresponding to the (223) crystal plane of the hexagonal phase Cs_4_PbBr_6_. It is worth mentioning that no impurities of another phase were found in 0D and 3D nanocrystals, respectively, which is consistent with the PL spectrum. Meanwhile, Fig. 3(d, e) shows the TEM images of the sample of 0D Cs_4_PbBr_6_ with 60 μl of water added in the toluene suspensions. It can be seen that the addition of water causes some perovskites to transform from hexagon to square shape. There are two types of morphology. One is aggregated square particles of an average size of 13 nm, as shown by the marked region in the upper right corner of Fig. 3(d). However, we were not able to measure the lattice spacing of the aggregated square particles. The second is a mixture of hexagon and square particles as shown in Fig. 3(e). The marked square and hexagon particles in (e) were analyzed in (f) for planar spacings of 3.12 Å and 2.95 Å, respectively. It was found that the hexagon particle has a size of 19 nm with a planar spacing of 2.95 Å corresponding to the (223) plane of 0D hexagonal Cs_4_PbBr_6_ crystal, similar to that in Fig. 3(i). Meanwhile, it was found that the square particle has a size of 15 nm with a planar spacing of 3.12 Å. We attribute this planar spacing to a diffraction angle of 28.586° of the 3D monoclinic CsPbBr_3_ (PDF#18-0364). However, this diffraction angle was not labeled to a particular crystal plane by PDF#18-0364. It is probably a combination of (111) plane and (002) plane of monoclinic CsPbBr_3_. Another possibility is that this lattice spacing corresponds to the plane (214) at a diffraction angle of 28.603° of a 0D hexagonal Cs_4_PbBr_6_. However, because the particle size is 15 nm, which is significantly smaller than the crystal size of Cs_4_PbBr_6_, and closer to the particle size of the square 3D CsPbBr_3_, together with the fact that the morphology is square-like, we believe that this 3.12 Å spacing belongs to the 3D monoclinic CsPbBr_3_ phase rather than the 0D hexagonal Cs_4_PbBr_6_ phase.

The smaller 0D Cs_4_PbBr_6_ after water addition in Fig. 3(d) indicates the partial dissolution of the original, larger 0D Cs_4_PbBr_6_ before water addition in Fig. 3(g). The partial dissolution of 0D Cs_4_PbBr_6_ led to the crystallization of 3D CsPbBr_3_ giving rise to the photoluminescence properties of the 0D/3D mixture phase.

**Figure 4:**
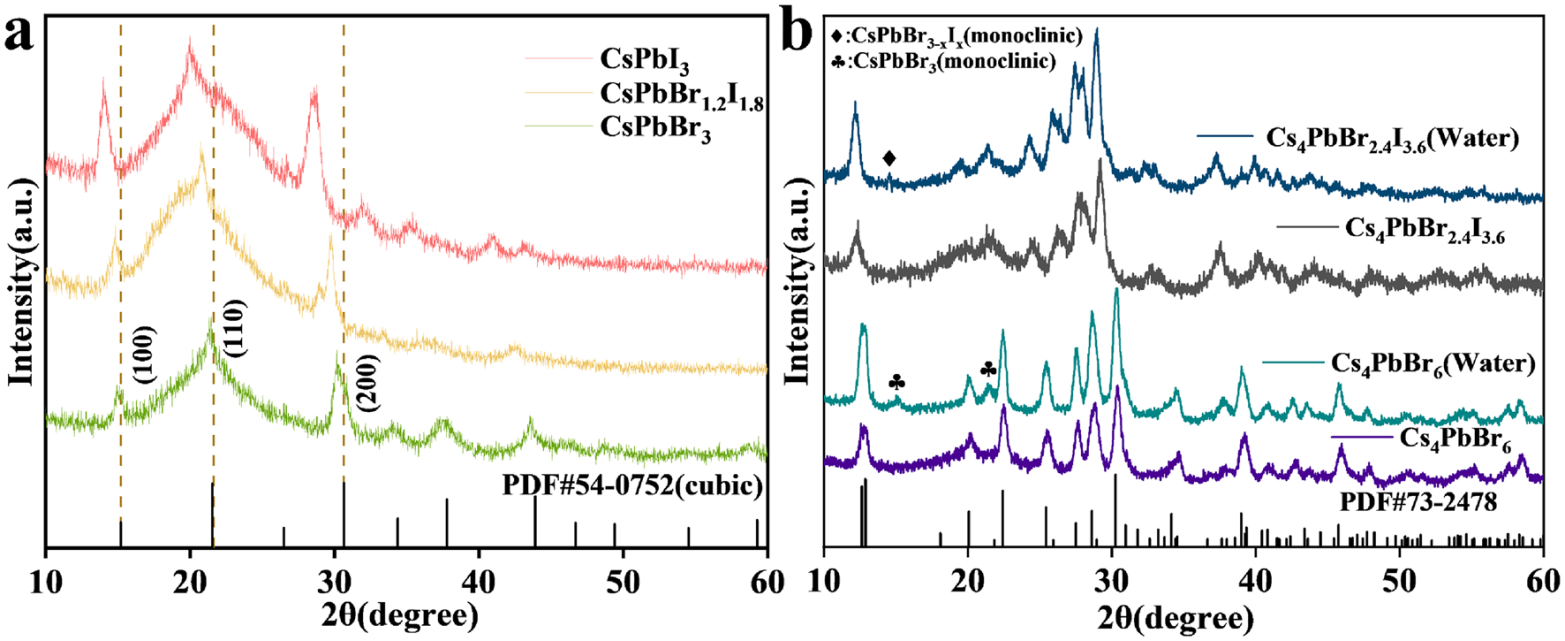
(a) XRD pattern of the CsPbX_3_ (*X* = Br, I, Br/I) and (b) XRD patterns of Cs_4_PbBr_6_ and Cs_4_PbBr_2.4_I_3.6_ nanocrystals and their changes upon the addition of water. Plum blossom symbol: Monoclinic CsPbBr_3_. Diamond: Monoclinic CsPbBr_3−*x*_I_*x*_ with *x* > 1.8.

To examine the crystal structure of the synthesized perovskites, we used XRD to analyze 0D and 3D perovskite nanocrystals. Figure 4(a) is the XRD pattern of CsPbBr_3_, CsPbBr_1.2_I_1.8_, and CsPbI_3_ nanocrystals, respectively. The vertical lines indicate the standard card of cubic phase CsPbBr_3_ (PDF#54-0752). Since the experimental method used the high-temperature synthesis method, all nanocrystals exhibit cubic phase structure. The main characteristic peaks of the CsPbBr_3_ spectrum appear at 15.1°, 21.5°, and 30.6°, corresponding to (100), (110), and (200) of the cubic phase of CsPbBr_3_. Compared with CsPbBr_3_, the XRD patterns of CsPbBr_1.2_I_1.8_ and CsPbI_3_ show a systematic shift to the left, in particular, the main characteristic peaks of CsPbI_3_ at 14.4°, 20.2°, and 28.8°, respectively, corresponding to the cubic (100), (110), and (200) phase of CsPbI_3_ [35]. The main reason for the shift to the left is the lattice expansion caused by the substitution of Br by I. Figure 4(b) shows the XRD patterns of Cs_4_PbBr_6_ and Cs_4_PbBr_2.4_I_3.6_ nanocrystals before and after the addition of water. The vertical lines show the hexagonal phase Cs_4_PbBr_6_ (PDF#73-2478). It can be seen from the diffraction pattern of Cs_4_PbBr_6_ that the main characteristic peaks at 12.8°, 22.4°, 25.4°, 28.6°, and 30.2° correspond to the (100), (300), (024), (214), and (223) crystal planes of the hexagonal crystal system Cs_4_PbBr_6_. The addition of water did not destroy the diffraction peak of Cs_4_PbBr_6_ nanocrystals but created new peaks at 15.2° and 21.4° (Plum Blossom symbols), which we speculate corresponding to the (100) and (110) crystal planes of the monoclinic phase CsPbBr_3_ (PDF#18-0364), indicating that the addition of water to Cs_4_PbBr_6_ created a mixture of Cs_4_PbBr_6_ and CsPbBr_3_. In other words, the addition of water dissolved some Cs_4_PbBr_6_ and precipitated new CsPbBr_3_ phase as shown by Wang et al. [18]. This behavior is also consistent with the results of TEM study shown in Fig. 3. Similar to the 3D perovskite system, the XRD pattern of 0D Cs_4_PbBr_2.4_I_3.6_ shows the addition of larger I ions causes the lattice expansion, resulting in a leftward shift of the diffraction angle relative to that of the Cs_4_PbBr_6_. After adding water to Cs_4_PbBr_2.4_I_3.6_, a new characteristic peak appeared at 14.6° (Diamond symbol), which is close to the representative monoclinic phase CsPbBr_3_ (100) that appeared at 15.2° during the water treatment of Cs_4_PbBr_6_. One possibility is that the 14.6° peak belongs to a 3D CsPbBr_3−*x*_I_*x*_ crystal produced by the dissolved Cs_4_PbBr_2.4_I_3.6_ when it meets water and the substitution of Br by I causes the peak position to shift from 15.2 to 14.6°. Regarding the unknown *x* value, we compare the PL behaviors in Fig. 1(c) of Cs_4_PbBr_2.4_I_3.6_ with water treatment and CsPbBr_1.2_I_1.8_. Since the PL spectra of Cs4PbBr_2.4_I_3.6_ with water treatment and CsPbBr_1.2_I_1.8_ are very different, we concluded that the unknown *x* > 1.8. In addition, since CsPbI_3_ has relatively poor stability and will degrade rapidly [16], we speculate that the photoluminescence of 0D Cs_4_PbBr_2.4_I_3.6_ is the result of the combined effect of CsPbBr_3_ precipitation with *x* CsI doping during the recrystallization process.

**Figure 5:**
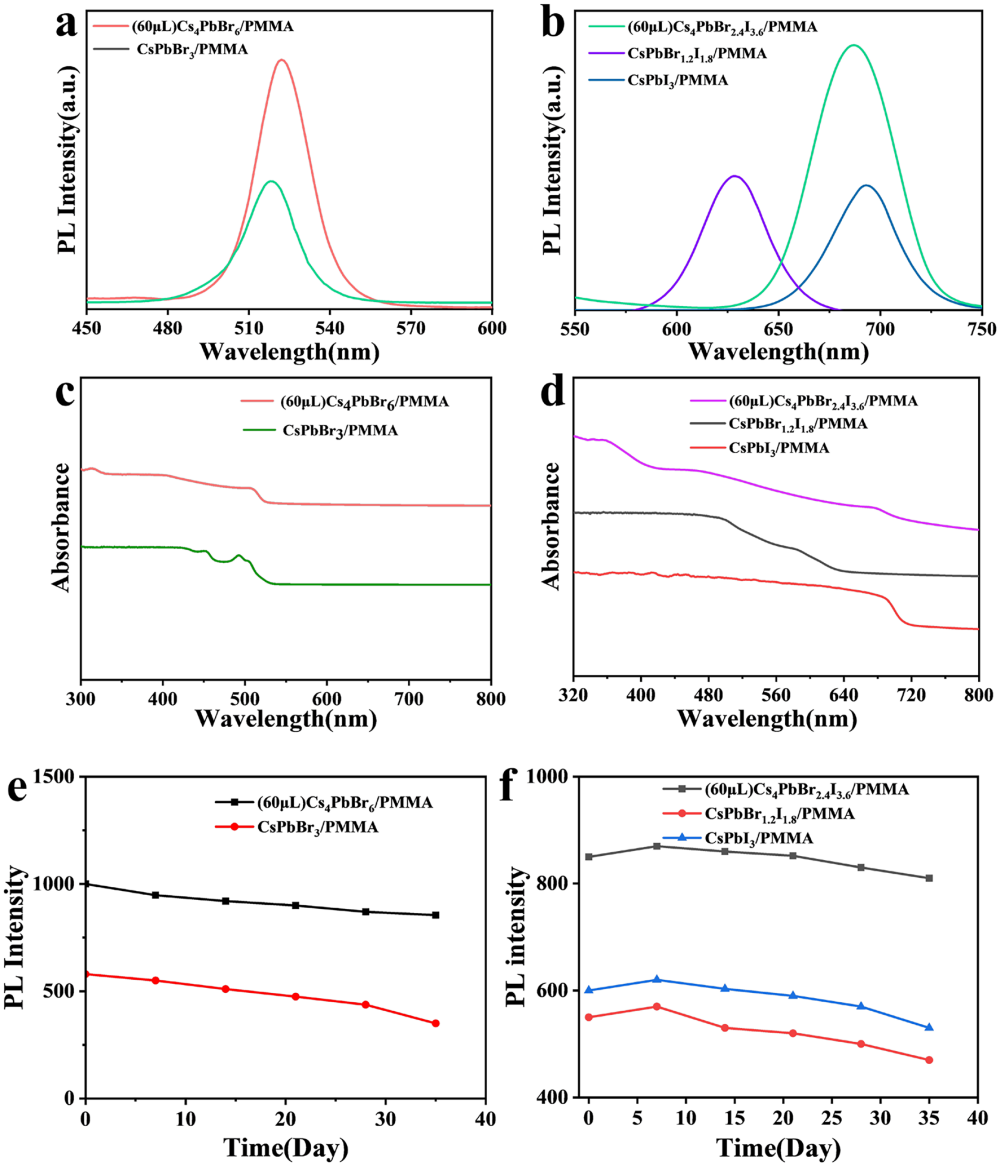
(a) PL spectra; (c) UV–vis spectra, (e) Cs_4_PbBr_6_/PMMA and CsPbBr_3_/PMMA composite films; (b) PL Spectra; (d) UV-absorption spectra; (f) PL stability over 35 days of Cs_4_PbBr_2.4_I_3.6_/PMMA, CsPbI_3_/PMMA, and CsPbBr_1.2_I_1.8_/PMMA composite films.

Figure 5(a) shows the PL of Cs_4_PbBr_6_/PMMA and CsPbBr_3_/PMMA composite films. The PL intensity of Cs_4_PbBr_6_/PMMA composite films compared to CsPbBr_3_/PMMA composite films is enhanced, and the position of the photoluminescence peak remains around 520 nm [36]. This behavior is consistent with the picture that due to the addition of water in toluene, part of the Cs_4_PbBr_6_ in toluene was dissolved and recrystallized into CsPbBr_3_ and protected by the PMMA matrix. The slight difference in PL peak position is likely because the size of the quantum dots may vary during the recrystallization process. Figure 5(b) shows that the photoluminescence peak of the 3D CsPbBr_1.2_I_1.8_/PMMA composite film was at 630 nm, while the position of the luminescence peak of 687 nm of the 0D Cs_4_PbBr_2.4_I_3.6_/PMMA composite film with 60 μl of water after 48 h was close to the 693 nm of CsPbI_3_/PMMA composite films. Such a large peak wavelength difference indicates that the Br/I ratio could not be maintained during the recrystallization process resulting in a change of the Br:I ratio because of water on the CsPbBr_1.2_I_1.8_ crystal [37]. Figure 5(c) shows the Cs_4_PbBr_6_/PMMA composite UV–vis peak appeared at 313 nm, [16] which is due to the increase in the band gap caused by the shift of local ^6^S_1/2_ → ^6^P_1/2_ in a single [PbX_6_]^4−^ octahedron isolated by Cs, indicating the existence of the Cs_4_PbBr_6_ phase [38]. The appearance of the same absorption peak in CsPbBr_3_ at 525 nm also confirmed that 60 μl of water resulted in the appearance of CsPbBr_3_. Figure 5(d) shows the absorption peak of Cs_4_PbBr_2.4_I_3.6_/PMMA composite films of 355 nm was offset by 15 nm of the 370 nm peak of Cs_4_PbI_6_ due to the increased band gap from Br doping. Meanwhile, the absorption peak of CsPbI_3_/PMMA composite films was around 690 nm. Figure 5(e, f) shows the PL change as a function of time of 0D and 3D perovskite/PMMA composite films in water over 35 days. It can be observed that the films have good water stability, which is likely brought about by the bonding of C=O with Pb^2+^ in the perovskite nanocrystals [39].

**Figure 6:**
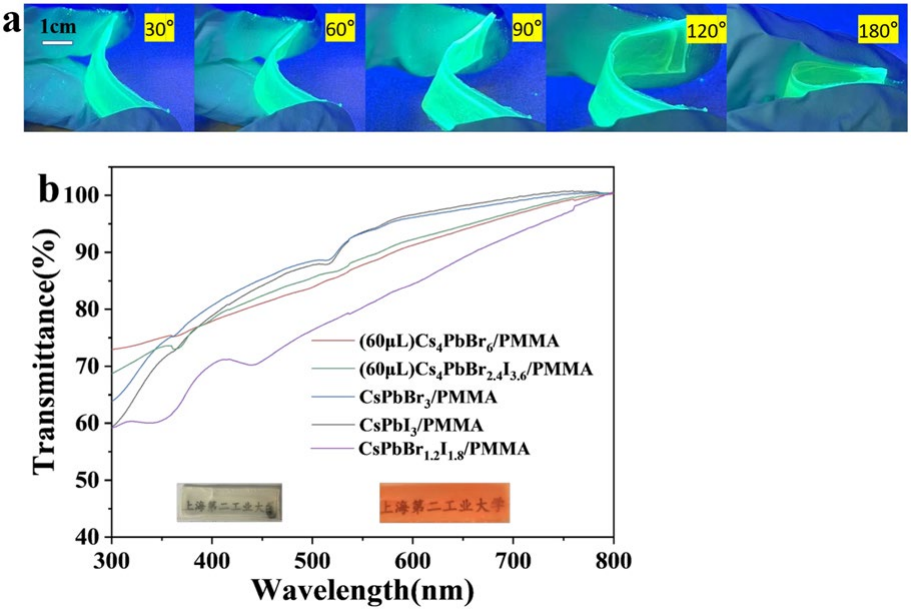
(a) Flexibility test of composite films; (b) transmittance as a function of wavelength; inset: Cs_4_PbBr_6_/PMMA (left) and Cs_4_PbBr_2.4_I_3.6_/PMMA (right) recrystallized by adding 60 μl water.

One possible application of composite films is white-light LED (WLED). We intend to create flexible WLED, because it will have a wider usage in the LED market. The flexibility of the composite film determines the mechanical properties of the WLED. In this regard, we analyzed the mechanical properties of composite films. As shown in Fig. 6(a), the composite films were bent from 30 to 180°, and it was found that they retained photoluminescence after bending. Furthermore, they can be restored after releasing the bending force without changing the luminescent properties. Meanwhile, good transmittance can reduce the power consumption of LED devices. Figure 6(b) shows the transmission spectrum of the composite films. Cs_4_PbBr_6_/PMMA composite films show a transmittance of > 60% in the wavelength range from 300 to 800 nm. Figure 6(v) also shows the pictures of a transparent Cs_4_PbBr_6_/PMMA composite film and that of the Cs_4_PbBr_2.4_I_3.6_/PMMA films. Both were placed above the text of Shanghai Polytechnic University. The text can be clearly seen illustrating their high transparency, indicating that the LED device prepared from them will have good transparency.

**Figure 7:**
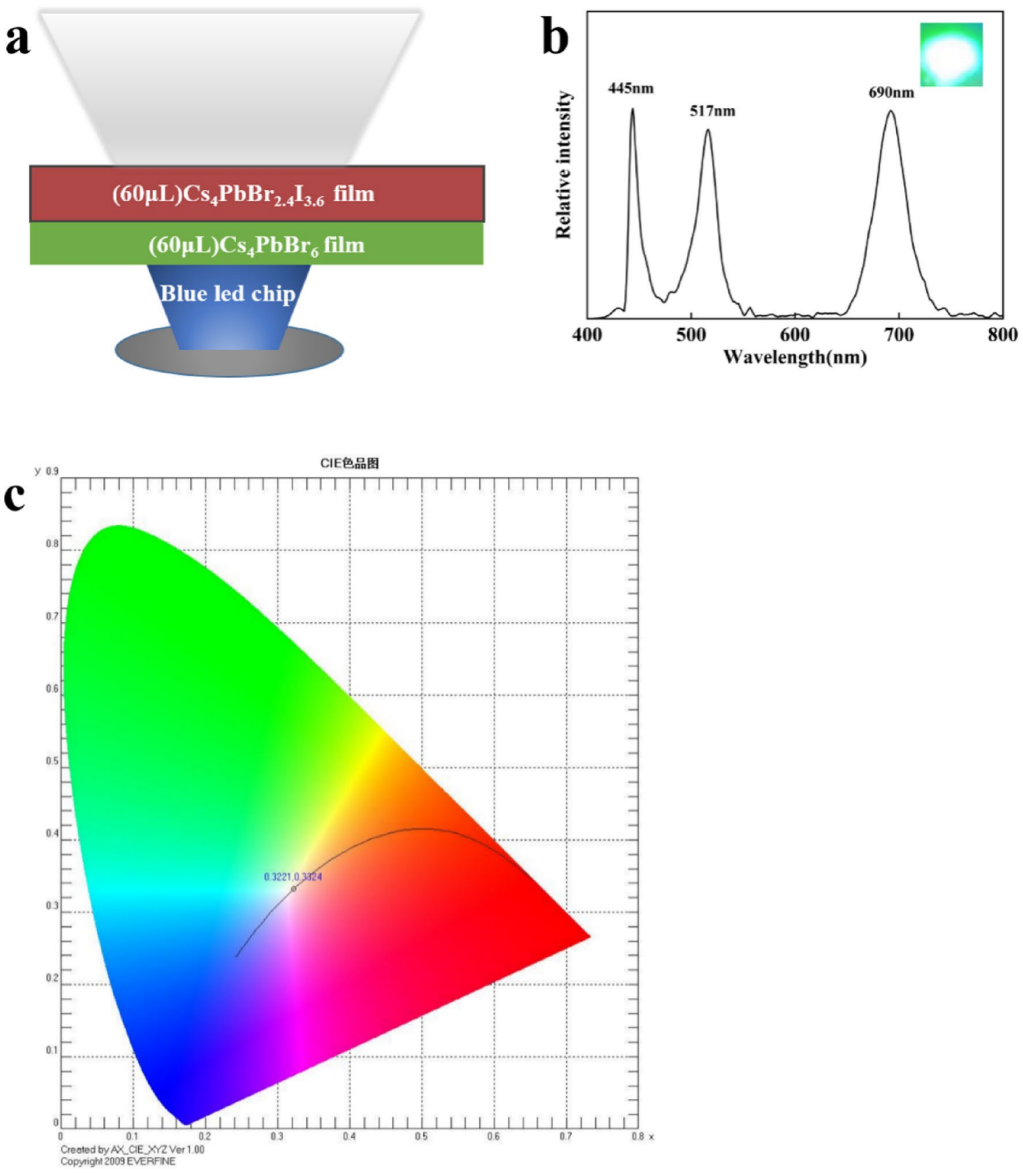
(a) Analog schematic diagram of white LED; (b) the emission spectrum of the white LED of the white LED (the illustration is a physical photograph of the white LED); (c) CIE chromaticity diagram of white LED.

Good flexibility and stability against water and light will give good performance of waterproof WLED devices. As shown in Fig. 7(a), we stacked Cs_4_PbBr_2.4_I_3.6_/PMMA and Cs_4_PbBr_6_/PMMA composite films sequentially on a blue LED with a current of 20 mA, sealed by black silicone and cured at room temperature for 30 min to obtain a WLED device. Figure 7(b) shows the corresponding luminescence peak positions of 445 nm, 517 nm, and 690 nm in blue, green, and red, respectively, and the inset is an LED white luminescence picture. Figure 7c is the CIE color diagram of the device, and the LED chromaticity coordinates produced are (0.3221, 0.3324) falling near the white-light area, with color rendering index 74.77 and a color temperature of 6997 K.

## Conclusion

We synthesized 0D inorganic perovskite Cs_4_PbX_6_ (X = Br, Br/I) suspension in toluene and created photoluminescence by adding a small amount of water in toluene. The 0D Cs_4_PbX_6_ (X = Br, Br/I) suspensions in toluene were mixed with a small amount of water and PMMA dissolved in toluene to prepare 0D Cs_4_PbX_6_/PMMA composite films. The prepared composite films have higher PL, stability, transparency, and transmittance than that of 3D CsPbX_3_/PMMA composite films prepared separately. Moreover, the flexible white-light LED device molded by composite films illustrated good luminescence performance with chromaticity coordinates of (0.32, 0.33), color rendering index 74.77, and color temperature of 6997 K.

## Data Availability

The data that support the findings of this study are available upon reasonable request and will be contained in the Master thesis of Mr. Yuang Ji at Shanghai Polytechnic University, 2024.
